# A Case of Hematemesis

**Published:** 1852-12

**Authors:** G. M. Bennet

**Affiliations:** Smithsville, Indiana


Art. III.—*A Case of Hematemesis.* By Dr. G. M. Bennet, of Smithsville, Indiana.

A. M., aged 52 years, living in a malarious region, temperate, a laborer, who had previously enjoyed good health, was seized with a chill on the 6th of October, after a few days of indisposition. I saw him in the fever which succeeded, but did not prescribe for him, as it was his wish to treat his own case. He took some purgative pills of a drastic nature, and a little quinine, eating during the day a piece of pickled cucumber. Soon after the pills began to operate he was attacked with nausea and vomiting, and it was at once perceived that he was throwing up blood from his stomach. Blood also passed from his bowels.

Oct. 7th. About 3 o'clock, p. m., I saw him, when he was in the following condition: He was vomiting blood at intervals of fifteen minutes, and passing it from his bowels; great thirst; tenderness in the epigastrium; extremities cold; breathing laborious or gasping; pulse scarely perceptible at the wrist; extreme restlessness. I prescribed cold drinks instead of the hot stimulants that had been given; cupped freely over the stomach, and followed the cupping with fomentations; and used sinapisms to the extremities. Still the paroxysms of nausea and vomiting continued to recur at short intervals, during which large quantities of blood were evacuated upwards and downwards. The blood discharged in vomiting was of the bright arterial hue; that from the bowels was less florid. The vomiting afforded only partial relief to the nausea, and the stomach continued to be oppressed.

Under these circumstances, I determined to try the effects of an emetic, and accordingly administered a full dose of ipecacuanha in a glass of cold water.

Under the operation of the emetic his pulse rose and the symptoms all improved. I gave him then two grains of the acetate of lead, and half a grain of sulphate of morphia.

In a short time the symptoms were as alarming as ever, and I resolved to repeat the emetic, encouraged by its good effects in the first instance. The effect was decided. His pulse rose, and the patient felt so much relieved that he began to speak of recovering. The lead and morphia were again given, and after a few hours the emetic was given the third time.

No hematemesis occurred after the last emetic, and his circulation became good. I gave him then a dose of calomel combined with morphia, which acted well on his bowels, bringing away large quantities of feces stained with blood.

8th. Patient remained comfortable till evening, when the nausea and sense of sinking returned; his extremities again grew cold, and he complained of tenderness in the abdomen, in the region of the descending colon; cups and fomentations were applied over the seat of abdominal tenderness, sinapisms and frictions to his extremities. The stomach again grew excessively irritable, and everything taken into the stomach was quickly rejected. Opiates, stimulants, and astringents were administered with only very slight advantage.

9th. The same state of things as that which prevailed yesterday still continues. The paroxysms of vomiting return hourly, or oftener; his thirst is very great, and his bowels are moved as often as he vomits; extreme restlessness, with suffocative breathing; the matter from his bowels has the appearance of dirty water, almost destitute of feculent matter. As the only thing that seemed to benefit the patient at all was the emetic, I determined to have recourse to it once more, and accordingly administered a dose of ipecac. in a little warm water.

The first influence of the emetic was alarming—he seemed to sink under its action; but reaction soon came on, and for some hours the patient was more comfortable. At the end of this time the spontaneous vomiting returned, with the serous purging; his extremities grew cold as high as the elbows and knees, and he was pulseless at the wrists. Wine and opium were freely given, and friction of the extremities was vigorously employed. Applied a blister to the epigastrium.

10th. Patient a little better. Blistèr has drawn well. The symptoms of yesterday persist, but in a mitigated degree. His stomach will retain nothing but the wine and opium, which are freely given. The opium does not narcotize him. Appearance of the patient much that of a man in the collapse of cholera.

Evening. A change is observable in the character of the alvine dejections, which are slightly feculent, with a tinge of bile, as if the calomel which I gave the day before, and supposed he had thrown up, might be producing some effect. Wine and opium to be continued.

11th. Patient better; stomach more tranquil, discharges from bowels still not satisfactory. Ordered a full dose of calomel and morphia.

The operation of the calomel was good, and the discharges favorable. His kidneys also began to act; his circulation was improved and nearly natural, except when affected by nausea. Stomach will retain no nourishment; little appetite; much thirst. Continue the stimulants and the opiate.

12th. Bowels have been quiet for some hours, and the patient is more comfortable. To-day he has some fever. This fever continued for a few days, being of a typhoid type; but it was not obstinate, and the patient went on improving, and is now well.

I am not sure that I understand the character of this case, but suppose that the affection was of malarious origin. The subject had suffered with chills, and his disease was probably a masked intermittent—one of the *febres intermittentes sub forma larvata*, of the old writers. A congestion of the portal circle induced by the malarial poison might naturally find vent in hemorrhage from the stomach.

I am the more inclined to this view of the subject from the fact that I have heard of the occurrence of a case similar to the foregoing in the practice of a medical friend. This patient was also a resident of a malarious district, and had been affected with the symptoms of an obscure intermittent. The quantity of blood discharged by emesis was very great—a chamber-pot was nearly filled, and the bed-clothing was saturated with blood. The hemorrhage recurred three or four times, at intervals of two, four, and six days, prostrating the patient to the last degree. He however recovered.

It is true that these cases, if cases of "intermittent fever," were very obscure, but they seem to me to bear more of that character than any other.

OCTOBER 25, 1852.

